# Effect of oxygen vacancy and Si doping on the electrical properties of Ta_2_O_5_ in memristor characteristics

**DOI:** 10.1038/s41598-023-43888-z

**Published:** 2023-10-03

**Authors:** Md. Sherajul Islam, Jonghoon Lee, Sabyasachi Ganguli, Ajit K. Roy

**Affiliations:** 1 Materials and Manufacturing Directorate, Air Force Research Laboratory, Wright-Patterson Air Force Base, Dayton, OH USA; 2 Spectral Energies, LLC, Dayton, OH USA; 3 ARCTOS Technology Solutions, Dayton, OH USA

**Keywords:** Electrical and electronic engineering, Theory and computation

## Abstract

The resistive switching behavior in Ta_2_O_5_ based memristors is largely controlled by the formation and annihilation of conductive filaments (CFs) that are generated by the migration of oxygen vacancies (OVs). To gain a fundamental insight on the switching characteristics, we have systematically investigated the electrical transport properties of two different Ta_2_O_5_ polymorphs ($\epsilon$-Ta_2_O_5_ and λ-Ta_2_O_5_), using density functional theory calculations, and associated vacancy induced electrical conductivity using Boltzmann transport theory. The projected band structure and DOS in a few types of OVs, (two-fold (O_2f_V), three-fold (O_3f_V), interlayer (O_IL_V), and distorted octahedral coordinated vacancies (O_ε_V)) reveal that the presence of O_IL_V would cause Ta_2_O_5_ to transition from a semiconductor to a metal, leading to improved electrical conductivity, whereas the other OV types only create localized mid-gap defect states within the bandgap. On studying the combined effect of OVs and Si-doping, a reduction of the formation energy and creation of defect states near the conduction band edge, is observed in Si-doped Ta_2_O_5_, and lower energy is found for the OVs near Si atoms, which would be advantageous to the uniformity of CFs produced by OVs. These findings can serve as guidance for further experimental work aimed at enhancing the uniformity and switching properties of resistance switching for Ta_2_O_5_-based memristors.

## Introduction

Of late, tantalum pentoxide (Ta_2_O_5_) has been extensively studied for its potential applications in memristors due to its high dielectric constant, high breakdown voltage, and good thermal stability^1–13^. One of the key advantages of Ta_2_O_5_ as a memristor material is its ability to undergo resistive switching (RS), a phenomenon in which the resistance of the material can be switched between high and low states by applying an electric field. Ta_2_O_5_ memristors, like other comparable metal oxides, are important due to their potential to revolutionize memory and computing technologies, facilitate new forms of artificial intelligence, curtail energy consumption, and empower the development of intricate and complex systems^8,9,14–17^. Usually, the RS phenomenon in Ta_2_O_5_ is attributed to the creation and disruption of conductive filaments (CFs) within the material, which can be controlled by the applied voltage and the presence of oxygen vacancies (OVs)^18–20^. However, the formation and characteristics of CFs in Ta_2_O_5_-based memristors can be complicated and greatly contingent on a number of variables, leading to several challenges in their development and optimization. Attaining consistent and replicable filament formation stands out as a primary obstacle. The formation and stability of CFs may exhibit significant susceptibility to the device configuration, the conditions of deposition, and the materials of the electrode, thereby posing challenges in attaining uniform and foreseeable performance. Hence, optimizing the device fabrication process and materials is crucial for achieving reliable and reproducible filament formation.

Earlier studies demonstrated that OVs are known to act as nucleation sites for the formation of CFs in Ta_2_O_5_ memristors^18–21^. Besides, OVs affect the stability of the CFs, which in turn can affect the endurance and retention of its resistive states, crucial to memristor’s performance reliability. OVs can also affect the switching dynamics of the memristor, including the voltage threshold, switching speed, and noise level. In this context, several studies have investigated the relationship between OVs and the electrical conductivity of Ta_2_O_5_^22–26^. For example, research has shown that OVs can increase electrical conductivity by creating additional charge carriers and defect states. The concentration and distribution of OVs can also affect the electrical conductivity by altering the material's band structure and carrier mobility. To the best of our understanding, the switching properties of Ta_2_O_5_ based memristor are correlated with the homogeneity of the material's nanoscale CFs. The ability of Ta_2_O_5_-based memristor to generate CFs may be attributed to the electric field-driven production and spread of OV. However, the existence of many disorders in the deposited resistive layer, the interaction of the OV with the dopant, and the difficulties in accurately defining the RS processes have made progress in this area difficult. To limit material trials and clarify the impact on system operation, it is helpful to have a theoretical knowledge of these defects. Therefore, it is critical to investigate how different dopants affect OV and how Ta_2_O_5_ -based memristor performance be improved.

On the other hand, depending on the fabrication method and conditions, Ta_2_O_5_ can exist in different crystal structures such as α-Ta_2_O_5_, β-Ta_2_O_5_, and ε-Ta_2_O_5_ phases^27–39^ that can affect the performance of the memristor. The most commonly used structure of Ta_2_O_5_ is the amorphous phase^40^. This is because amorphous Ta_2_O_5_ can undergo a reversible RS phenomenon that is essential for the operation of memristors. Other structures of Ta_2_O_5_, such as the orthorhombic, tetragonal, monoclinic, and cubic phases, may also be used for memristor applications^32,34,37^. However, these structures may require additional processing steps, such as annealing or doping, to achieve the desired properties for memristor operation. Moreover, a hidden polymorph namely called as lambda phase (λ-Ta_2_O_5_) can also exist^30^. Different crystal structures of Ta_2_O_5_ may exhibit different resistive switching behaviors because the breakdown voltage should be different for each structure (usually proportional to the square of the bandgap energy), which can make it challenging to optimize the performance of the device. To fully understand the switching mechanism of Ta_2_O_5_ in memristors, it is important to identify the mechanisms involved in the switching behavior, such as materials crystalline phase influencing oxygen migration and defect formation, and to understand how these mechanisms are affected by different experimental conditions stated above.

In this work, instead of studying all Ta_2_O_5_ crystalline phases we discussed above, we limit our work investigating the vacancy induced electrical transport properties of orthorhombic and lambda phase Ta_2_O_5_, due to their good agreement with the experimentally observed electrical band gap of amorphous Ta_2_O_5_, using density functional theory investigations. The effect of OV on the electrical conductivity of Ta_2_O_5_ has been calculated using Boltzmann transport theory. Chemical doping, combined with OVs, is known in many cases to impact the performance of Ta_2_O_5_ memristors and help optimize key parameters such as the RS characteristics, endurance, retention, and variability. Although several molecules as dopant to Ta_2_O_5_ are feasible^41–43^, however, here only the combined effect of Si-doping and OV on electrical conductivity are investigated. To explore the local structural changes due to OV and doping effect, the formation energy, projected electronic band structure, and projected density of states have been calculated. This study can guide further experimental efforts aimed at enhancing the uniformity of RS and switching properties of Ta_2_O_5_-based memristor devices.

## Calculation methods

All the investigations have been conducted using the ab initio first-principles plane wave pseudopotential approach executed in the Vienna Ab initio Simulation Package (VASP)^44,45^. The electron exchange correlation is obtained using the generalized gradient approximation of the Perdew-Burke-Ernzerhof solid (GGA-PBEsol) functional^46^. The structure is optimized using the RMM-DIIS algorithm with the force convergence of 0.02 eV/Å (1 × 10^–5^ eV). Following relaxation, the electronic structures are characterized using a kinetic energy cutoff of 400 eV. The Brillouin-zone is sampled with a k-point mesh of 17 × 13 × 9 for the structural relaxations and all the electronic properties calculations. The electrical conductivity has been calculated using the Boltzmann transport theory as implemented in MedeA VASP. Using the Boltzmann transport theory^47^, one can represent the electrical conductivity tensor of a solid in terms of energy dependence as

1 $${\sigma }_{\alpha \beta }(E)=\frac{{e}^{2}}{{\Omega }_{C}}\sum_{k}\sum_{n}\left(-\frac{\partial f(E)}{\partial E}\right){v}_{kn}^{\alpha }{v}_{kn}^{\beta }{\tau }_{kn}$$

where *f*(E) denotes the Fermi function $f(E)=\frac{1}{{e}^{\beta (E-\mu )}+1}$, with *μ* being the chemical potential, $\beta =$ $\frac{1}{{k}_{B}T}$*.* ${\tau }_{kn}$ stands for the relaxation time, which relies on the band index (*n*), spin, and *k*-point and replicates phenomena such as electron–phonon scattering on the electronic states. ${v}_{kn}^{\alpha }$ represents the group velocity. The first derivative of the band energy (${\epsilon }_{kn}$) with respect to the appropriate Cartesian component ($\alpha$) of the *k*-vector yields the ${v}_{kn}^{\alpha }$ for each band (*n*) and *k*-point as

2 $${v}_{kn}^{\alpha }=\frac{1}{\hslash }\frac{\partial {\epsilon }_{kn}}{\partial {k}_{\alpha }}$$

The electrical conductivity can further be reduced by relating the so-called transport distribution^47,48^ as

3 $${\Xi }_{\alpha \beta }(E)=\frac{1}{{\Omega }_{C}}\sum_{k}\sum_{n}{v}_{kn}^{\alpha }{v}_{kn}^{\beta }{\tau }_{kn}\delta (E-{\epsilon }_{kn})$$

Thus, the electrical conductivity coefficient, ${\sigma }_{\alpha \beta }$ can be represented as

4 $${\sigma }_{\alpha \beta }={e}^{2}{\int }_{-\infty }^{\infty }dE(-\frac{\partial f(E)}{\partial E}){\Xi }_{\alpha \beta }(E)$$

To calculate electrical conductivity, the self-consistent field (SCF) charge density of the complete system is determined in the first stage with a k-point mesh of 9 × 5 × 5. Based on the SCF charge density obtained in the preceding phase, the Fermi surface and a band structure are then generated with a relatively fine and regular k-point grids of 17 × 13 × 9. The band structure acquired in the second phase is then used to compute the electronic transport characteristics using the BoltzTraP code^49^.

## Results and discussion

**Figure 1:**
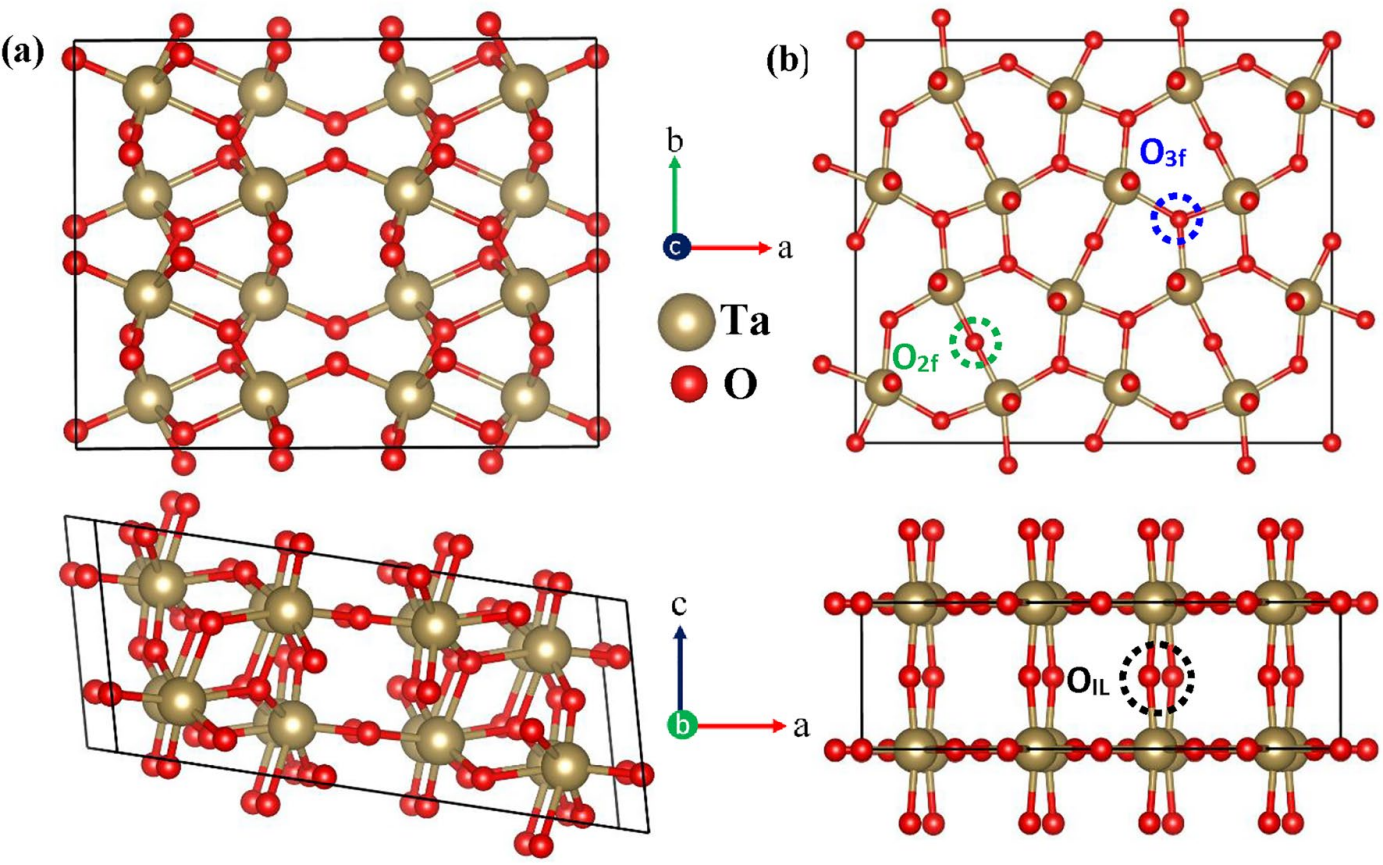
Structural model for (**a**) $\epsilon$-Ta_2_O_5_ and (**b**) λ-Ta_2_O_5_ phases. Upper and lower figures indicate the ‘ab’ and ‘ac’ plane views of $\epsilon$-Ta_2_O_5_ and λ-Ta_2_O_5_ structures, respectively. Green, blue, and black circles in (**b**) indicate twofold, threefold, and interlayer coordinated O atoms, which are denoted as O_2f_, O_3f_, and O_IL_, respectively.

**Figure 2:**
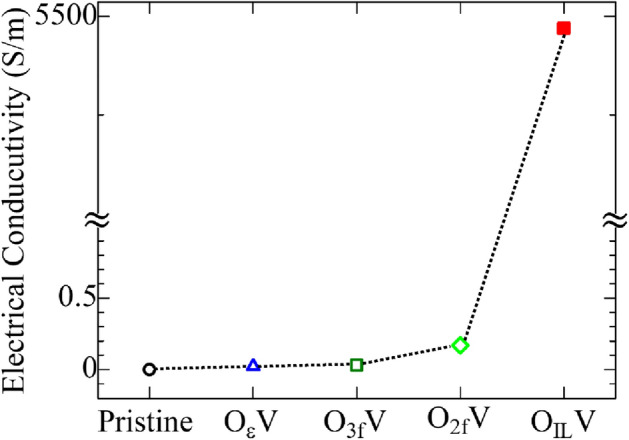
Electrical conductivity of different types of oxygen vacancies induced Ta_2_O_5_ at room temperature (300 K).

Among many different polymorphs of Ta_2_O_5_ that have been discovered, the β-Ta_2_O_5_ (orthorhombic space group P*mmm*)^35,39,50–53^ is the most studied polymorph because it is thought to be stable at low temperatures and experience a phase transition to α-Ta_2_O_5_ at relatively high temperatures (1630 K)^39^. Another common phase is $\epsilon$-Ta_2_O_5_ (space group C2/c)^36^, which can be produced using either a chemical method^36^ or a high-pressure synthesis^37^. The amorphous phase, the hexagonal or δ phase, the high-pressure-prepared phase, and the polymorph with a high number of OVs can also be detected by the X-ray particle diffraction observations^31–33,54^. In addition, λ-Ta_2_O_5_ has drawn a lot of attention recently due to its high structural stability and good agreement with the experimentally observed electrical band gap of amorphous Ta_2_O_5_^30^. We primarily concentrate on the last two demonstrative phases, specifically $\epsilon$-Ta_2_O_5_^37,55–58^ and λ-Ta_2_O_5_^19,59,60^, as they can be produced under comparatively favorable circumstances and are generally thought to be sufficiently stable for use in real-world applications, as compared to the other phases. It is noted that our examination of various Ta_2_O_5_ polymorphic structures is by no means comprehensive; instead, the primary focus of this article is on analyzing the impacts of varying OVs on certain electronic transport characteristics. $\epsilon$-Ta_2_O_5_ phase belongs to the monoclinic structure with the C2/c symmetry and λ-Ta_2_O_5_ phase belongs to the orthorhombic structure with the Pbam symmetry. In $\epsilon$-Ta_2_O_5_ crystal structure, each Ta atom has a distorted octahedral coordination environment, with six neighboring O atoms. On the other hand, three different O sites can be found in the λ-Ta_2_O_5_ structure: the doubly and threefold coordinated sites in the Ta_2_O_3_ plane, as well as the twofold coordinated site of the Ta-O-Ta interlayer chain, as shown in Fig. 1. The calculated lattice parameters of the optimized structures of both phases along with the experimentally obtained values are presented in Table 1. The optimized lattice parameters derived from PBEsol computations for both λ-Ta_2_O_5_ and $\epsilon$-Ta_2_O_5_ structures show excellent agreement with experiment and theoretical predictions (left column in Table 1)^30,37^, which also indicating that the PBEsol GGA functional performs well in forecasting structural characteristics of solid. In light of this, the PBEsol optimized structure could be used as a suitable beginning point for the ensuing electronic structure computations. The analyses start with the calculations of different types of OVs-induced electrical conductivity for two different phases of Ta_2_O_5_. In λ-Ta_2_O_5_ structure, three types of OVs are defined according to their coordination sites such as O_2f_V_,_ O_3f_V, and O_IL_V. The OV in $\epsilon$-Ta_2_O_5_ structure is represented by the O$\epsilon$V. We have taken 2 × 2 × 1 and 2 × 1 × 1 supercells for λ-Ta_2_O_5_ and $\epsilon$-Ta_2_O_5_ structures, respectively, which contains 16 Ta atoms and 40 O atoms for both structures. The estimated electrical conductivity for different types of OVs is shown in Fig. 2.

**Table 1:** Calculated structural parameters of $\epsilon$-Ta_2_O_5_ and λ-Ta_2_O_5_ phase.

Chemical formula	$\epsilon$-Ta_2_O_5_^37^	This work	λ-Ta_2_O_5_^30^	This work
Cell setting	Monoclinic	Monoclinic	Orthorhombic	Orthorhombic
Space group	C2/c	C2/c	Pbam	Pbam
a (Å)	12.7853	12.74156	6.25	6.216
b (Å)	4.8537	4.8200	7.40	7.338
c (Å)	5.5276	5.49353	3.83	3.798
α (º)	90	90	90	90.00
β (º)	90	90	90	90.00
γ (º)	104.264	104.22	90	90.00

The calculated results revealed that with the introduction of a single OV, the electrical conductivity tends to increase for all types of vacancy defects, although the increase of conductivity for all the vacancy types is not the same. O$\epsilon$V, O_2f_V, and O_3f_V type vacancy defects show a slight increase in conductivity compared to the pristine Ta_2_O_5._ In contrast, the O_IL_V defect shows a considerable increase in conductivity. In general, OVs can act as electron traps, leading to changes in the electronic structure of the material and altering its electrical conductivity. In the case of Ta_2_O_5_, OVs are expected to enhance the electrical conductivity of the material because the presence of OVs introduces electronic states within the band gap of Ta_2_O_5_, which can act as charge carriers and increase the conductivity. These electronic states are usually located closer to the conduction band, which means that electrons can easily move into these states and contribute positively to the conductivity. Experimental studies have confirmed that oxygen vacancies do indeed affect the electrical conductivity of Ta_2_O_5_. For example, Seki et al. found that annealing Ta_2_O_5_ films in reducing atmospheres (i.e., environments with a low oxygen partial pressure) resulted in a significant increase in the films’ electrical conductivity^61^. This was attributed to the creation of OVs in the material.

**Figure 3:**
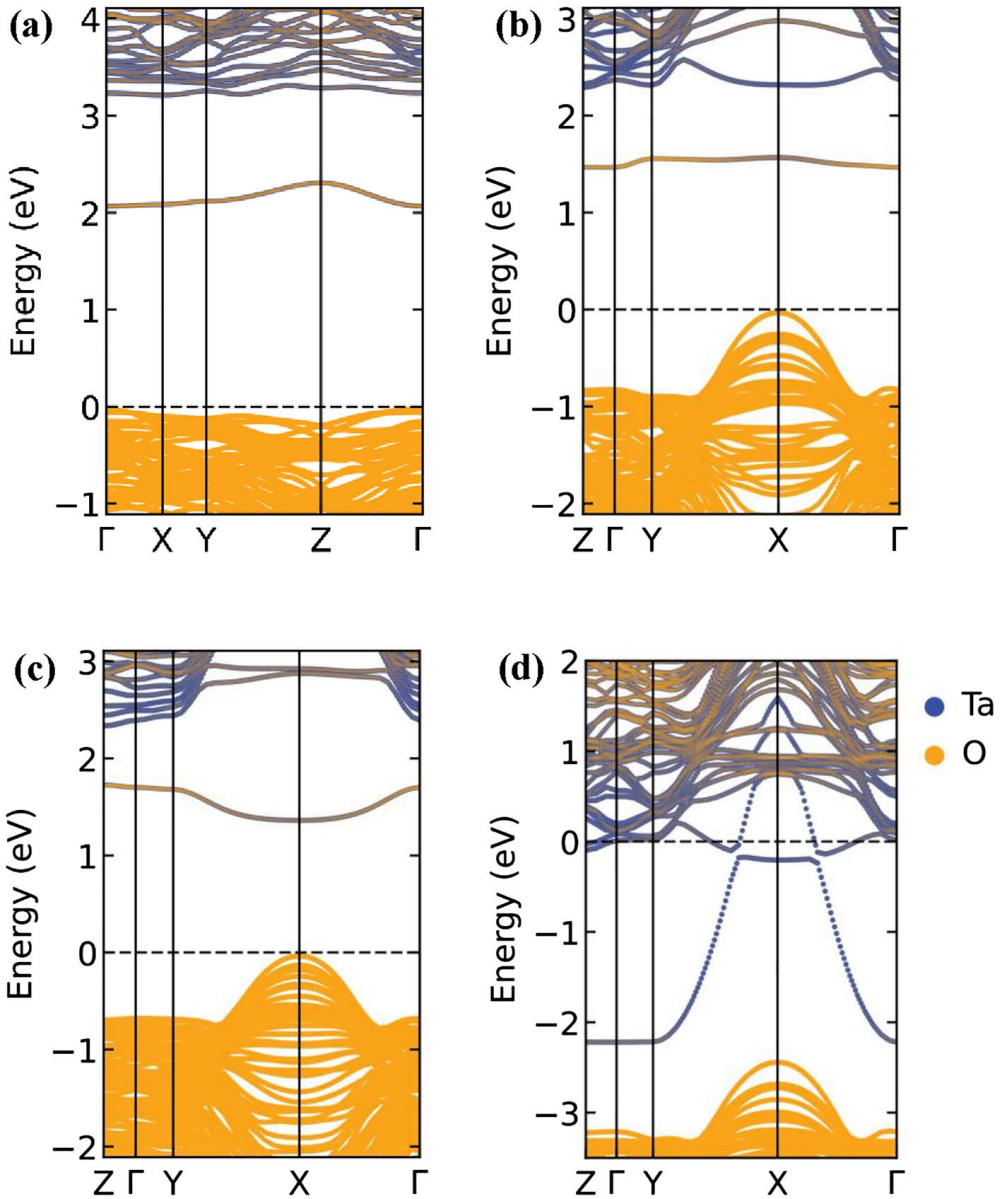
Projected electronic band structures of Ta_2_O_5_ with single (**a**) O$\epsilon$V in $\epsilon$-phase, and (**b**) O_2f_V, (**c**) O_3f_V, and (**d**) O_IL_V type defects in λ-phase. The reference energy level (dotted line) is touched with the valence band maximum for all types of OVs. Yellow and blue colors represent the contribution form O and Ta atoms, respectively.

To comprehend the vacancy induced electrical conductivity in both structures, we have calculated the electronic band structure for all these types of vacancies. The electronic band structure from PBEsol for pristine $\epsilon$-Ta_2_O_5_ and λ-Ta_2_O_5_ structures are presented in supplementary information (Figure S1). The $\epsilon$-Ta_2_O_5_ structure shows a larger band gap by pushing the conduction band minimum (CBM) to higher energy compared to the λ-Ta_2_O_5_ structure. An electronic gap of 3.18 eV and 2.16 eV is observed for $\epsilon$-Ta_2_O_5_ and λ-Ta_2_O_5_ structures, respectively. The obtained bandgap is well matched with the previous studies^19,30,37^. It is worth to mention, earlier investigations demonstrated that HSE functional may estimate a higher band gap compared to PBE functional^19,30^. However, our focus here is to understand how conductivity is changed due to OVs, and hence the PBEsol is justifiably an acceptable choice for this study. It is evident from the band structures that the O atoms form the valence bands, while the conduction bands are formed by the delocalized electrons of the Ta atoms, as shown in Fig. 3. As evident from the data in Fig. 3, the OVs in Ta_2_O_5_ can reduce the band gap of the material through the formation of defect states within the band gap. When an O atom is missing from the crystal lattice, there is a local change in the charge balance within the crystal, leading to the formation of electronic defect states. This defect state can act as an intermediate energy level between the valence and conduction bands, allowing electrons to be promoted across the bandgap more easily. This may cause an increase in the conductivity of the materials. However, the energy gap between the CBM and the defect band energy (DBE) is ~ 0.8 eV, and between the valence band maximum (VBM) and the DBE is ~ 2 eV (as shown in Fig. 3a–c). Both gaps are too large for the electron transition at room temperature (25 meV). The degree to which the bandgap is affected by OVs in Ta_2_O_5_ depends on the types of vacancies within the material.

Removing one O atom from O$\epsilon$, O_2f._, and O_3f._ coordinated sites do not occupy vacant conduction band states to render the system metallic; rather they confine in a mid-gap defect state, confined at the vacancy site as shown Fig. 3a–c. The defect states just lead to the appearance of additional electronic states within the bandgap, without necessarily changing the overall band structure of the material. The system is semiconducting in all instances because there is a gap between the highest occupied and lowest unoccupied states. The estimated energy differences between the conduction band and the defect states are 0.877 eV, 0.606 eV and 0.614 eV for O$\epsilon$V, O_2f_V, and O_3f_V type vacancy defects, respectively. On the other hand, the introduction of the O_IL_V defect results in a semiconductor-to-metal transition. The semiconductor to metal transition due to an O_IL_V type defect is characterized by the filling of the conduction band with electrons, resulting in a partially filled conduction band that is characteristic of metals, as shown in Fig. 3d. This leads to higher electrical conductivity for this type of vacancies. It is worth noting that the effects of OVs on the bandgap of Ta_2_O_5_ are complex and can be influenced by a variety of factors, including the crystal structure of the material, the nature of the OVs themselves, and the presence of other impurities or defects in the material. Therefore, a thorough understanding of the properties of Ta_2_O_5_ and its behavior in different environments is necessary to accurately predict and control its bandgap energy.

**Figure 4:**
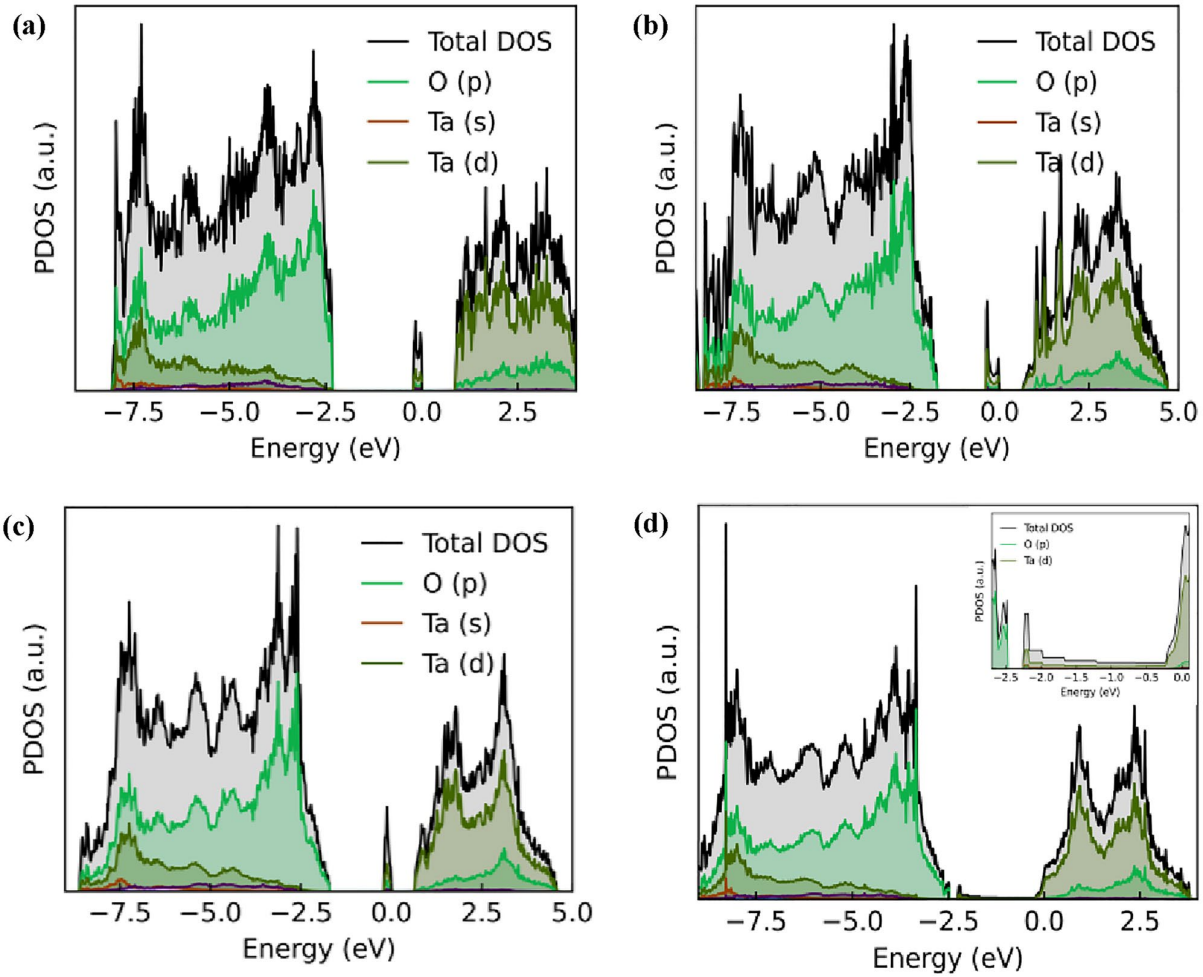
PDOS of Ta_2_O_5_ with single (**a**) O$\epsilon$V, (**b**) O_2f_V, (**c**) O_3f_V, and (**d**) O_IL_V type defects. Inset shows the zoom-in view of a broadened peak covering the entire gap region.

We have thus further assessed the projected density of states (PDOS) to get more information on the nature of the electronic transitions that occur in the material and the role of OV defects and impurities in the electronic structure. Figure 4 shows the PDOS for various types of OV defects for both λ-Ta_2_O_5_ and $\epsilon$-Ta_2_O_5_ phases, respectively. The presence of OVs in Ta_2_O_5_ significantly affect its PDOS due to the changes in the electronic structure caused by the introduction of defect states. It is observed from PDOS that the valence band is mainly composed of oxygen p-orbitals and the conduction band is mainly composed of Ta d-orbitals. The PDOS of Ta_2_O_5_ without any OVs typically shows a band gap between the valence band and the conduction band, as shown in supplementary information (Figure S2). When OVs are introduced into the Ta_2_O_5_ lattice, the electronic structure changes due to the formation of defect states within the band gap. OVs lead to the appearance of new peaks and features in the PDOS of both λ-Ta_2_O_5_ and $\epsilon$-Ta_2_O_5_ structures. In terms of specific PDOS contributions, the OVs in Ta_2_O_5_ can lead to an increase in the density of states near the Fermi level associated with the Ta 5d orbitals and the O 2p orbitals. This indicates that the electronic states associated with these orbitals are more strongly affected by the OVs.

**Figure 5:**
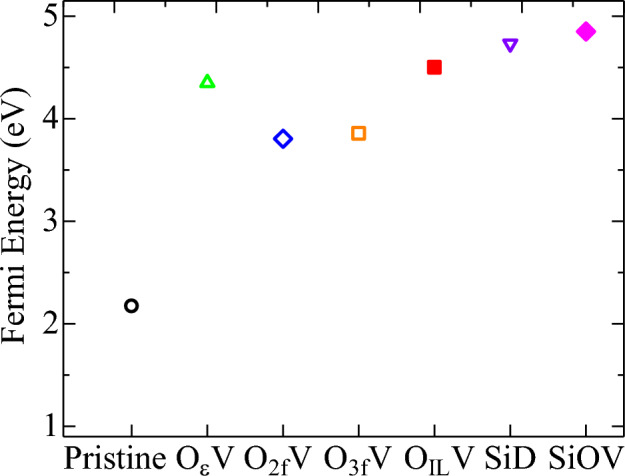
Changes of Fermi energy for different types of oxygen vacancies.

The number and position of the defect states depend on the location of the oxygen vacancies in the lattice. Although some sharp peaks are observed in the middle of the gap for O$\epsilon$V, O_2f_V and O_3f_V defects, a broadened peak covers the entire gap region for O_IL_V type defect, indicating the semiconductor to metal transition in this defect type. Moreover, it is observed that the Fermi level is shifted towards the conduction band with the introduction of OV. The Fermi level for semiconductor (insulator) at 0 K is simply at (VBM + CBM)/2. In Fig. 3a–c, the Fermi level should be at (CBM + DBE)/2 at 0 K because the highest occupied state is at the top of DBE, indicating that the Fermi level moves towards the conduction band for all types of OVs. The OV causes the Fermi level to move; therefore, the distribution *f*(E) in Eq. 4 changes with the OV. The integral sampling over E in Eq. 4 has the heavier weight of *f*(E) when E is near the Fermi level. Now, with OV, the Fermi level moved closer to the conduction band, which increased the weight *f*(E) for the conduction band. Furthermore, the Fermi level lies in the middle between DBE and CBM at 0 K, which further moves closer to CBM as temperature increases. The DFT calculated values of Fermi energy for λ and $\epsilon$-phase pristine structures are 2.17 eV and 2.008 eV, respectively, whereas these values are 4.351 eV, 3.805 eV, 3.856 eV, and 4.5009 eV for O$\epsilon$V, O_2f_V, O_3f_V and O_IL_V type defects, respectively, as shown in Fig. 5. The shifting of Fermi energy towards the conduction band leads to an increase in the number of free electrons available for conduction, which can increase the electrical conductivity of the material. When OVs are present, the oxygen 2p states near the CBM become empty, and some of the electrons from the Ta 5d states may transfer to these vacant oxygen states. This transfer of electrons leads to an increase in the Fermi energy level and a shift towards the CBM. The increase in electrical conductivity due to a Fermi level shift towards the conduction band is observed in many types of materials, including semiconductors, metals, and insulators^62–66^.

We have also estimated the formation energy (${E}_{fe}$) for each of the aforementioned defect types. The ${E}_{fe}$ of OVs in Ta_2_O_5_ is closely associated to the SET/RESET voltage and power utilization of the memristor during RS^19^, as it represents the defect-forming capability and the stability of defects. The voltage needed to move the device from a high-resistance state (HRS) to a low-resistance state (LRS) is known as the SET voltage, whereas the voltage needed to transfer the device from a LRS to a HRS is known as the RESET voltage. If the ${E}_{fe}$ of OVs is too low, it can result in a high concentration of vacancies, which can lead to poor endurance and retention characteristics. Therefore, it is important to optimize the ${E}_{fe}$ of OVs in Ta_2_O_5_-based memristors to achieve the desired SET/RESET voltage and power consumption while maintaining good endurance and retention characteristics. This can be achieved by tuning the material composition, crystal structure, and processing conditions to control the concentration and distribution of defects in the material. The ${E}_{fe}$ of OV can be evaluated using the expression^67–70^ as, $E_{fe} = E_{OV} - E_{pristine} - \sum n\varsigma$, where ${E}_{OV}$ and ${E}_{pristine}$ represent the total energy of the OV-defected and pristine structures, respectively. The number of atoms necessary to create OV is *n*. If n is greater than 0 then it is necessary to add atoms to the defect-free model, and if n is less than 0 then it is necessary to delete atoms from the pristine model. $\varsigma$ stands for the chemical potential of the associated atom. The value of $\varsigma$ for the oxygen atom is used in the calculation as 4.93 eV^19^. The calculated ${E}_{fe}$ for the O$\epsilon$V, O_2f_V, O_3f_V, and O_IL_V are 6.50 eV, 6.21 eV, 5.25 eV, and 4.40 eV, respectively, which is relatively high. However, this finding is in line with the earlier investigations^71^. Among the ${E}_{fe}$ calculated for all OV types, the O_IL_V shows the comparatively lowest value, indicating that this type of OV is more likely to be present in the system. While the generation and manipulation of specific types of OVs pose challenges, various processes can be employed to achieve this, including oxygen annealing or sputtering in controlled atmospheres during device fabrication, electroforming through the application of high-voltage pulses or voltage sweeps across the memristor, and the utilization of voltage or temperature variations.

**Figure 6:**
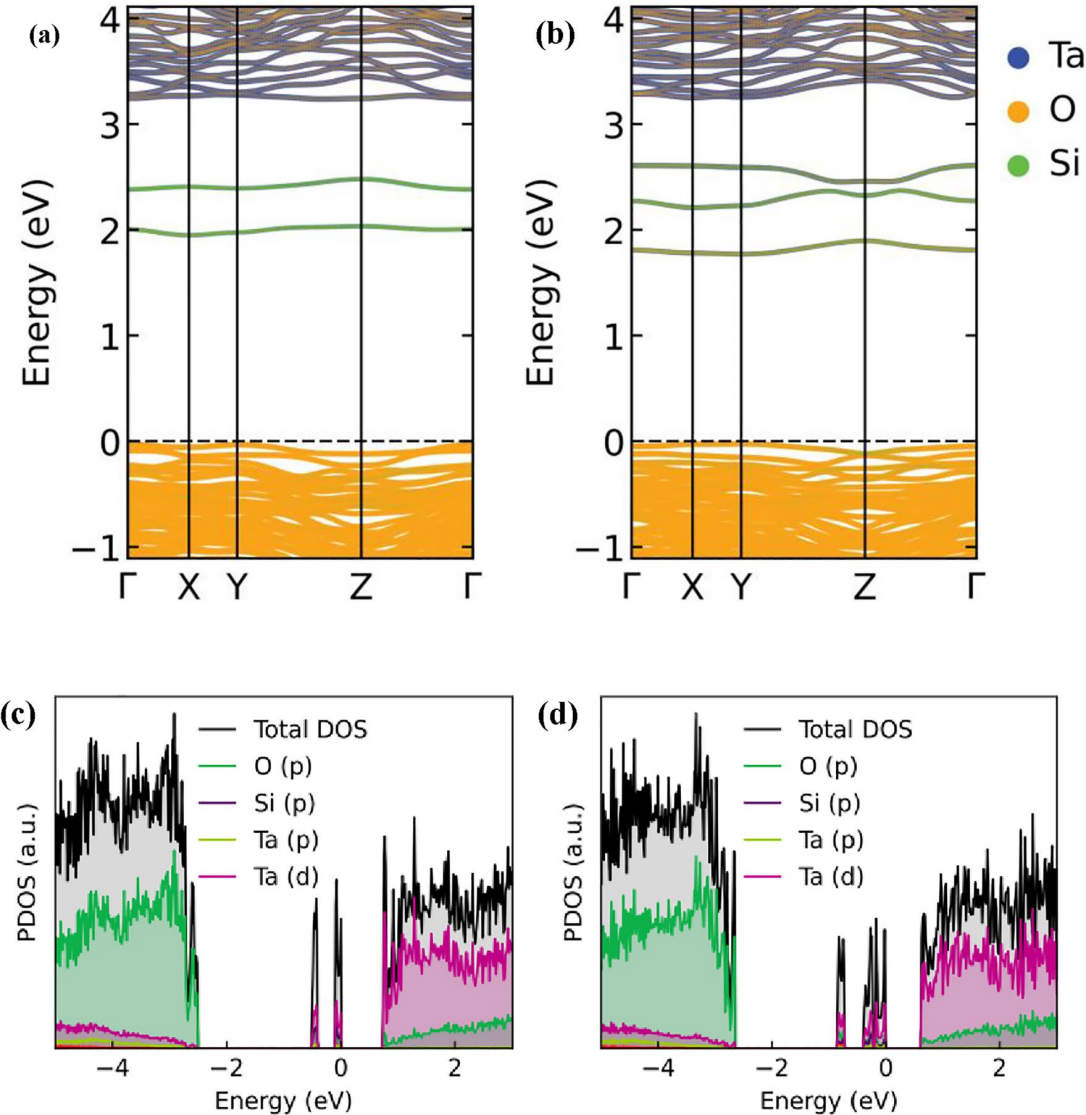
Projected electronic band structure of $\epsilon$-Ta_2_O_5_ structure with (**a**) Si-doping and (**b**) combined Si-doping and one OV. PDOS of $\epsilon$-Ta_2_O_5_ structure with (**c**) Si-doping and (**d**) combined Si-doping and one OV.

Finally, in search for lowering the high SET/REST voltage predicted above, we have investigated the doping effect and explored the combined doping and OV effect on the electrical properties in Ta_2_O_5_. To investigate the doping effect, we have considered $\epsilon$-Ta_2_O_5_ structure as a representational unit as its electrical properties are closer to the realistic applications. It is revealed that the doping of N atom in Ta_2_O_5_ structure decreases the band gap and follows the sequence of oxide > oxynitride > nitride, which is in good agreement with experimental results as well^58^. It is worthy to note that several studies have been performed in improving the electronic properties of Ta_2_O_5_-based resistive memristor devices using ionic doping technology. Some successful doping strategies include Ti doping^41^, which has been shown to produce soft collapse with high ON/OFF ratio in Ta_2_O_5_-RRAM devices. Al doping has also been found to reduce the forming voltage required for the device to switch states^42^. More recently, Zr doping has been shown to produce better switching performance in Ta_2_O_5_-RRAM^43^. However, it is important to note that metal dopants can also have negative effects on device performance. For example, metal dopants can cause field enhancement effects that would reduce the stability and uniformity of CFs produced by OVs as the number of cycle increases^72,73^. This can lead to deterioration in the high resistive state retention of the device, which can limit its overall performance and reliability. In contrast, the use of nonmetallic Si dopants in Ta_2_O_5_-RRAM devices may offer a promising approach for improving the performance and reliability of these devices, particularly in terms of maintaining uniform CFs and avoiding the negative effects of field enhancement^19^. We have thus replaced one O atom with one Si atom in $\epsilon$-Ta_2_O_5_ structure and then OVs are created by removing O atoms. The OV-induced projected band structure and projected density of states of Si-doped $\epsilon$-Ta_2_O_5_ are represented in Fig. 6. An earlier study revealed that the OV in Si-doped Ta_2_O_5_ may have negligible effect on conductivity as there is no defect state in the band gap and the defect energy level is close to the VBM^19^. However, our calculated projected band structures and PDOS clearly state that the introduction of OVs in Si-doped Ta_2_O_5_ leads to the formation of defect states near the conduction band edge, which can participate in electronic transport. There are possibly two factors contributing to the different simulation outcomes seen in the earlier study conducted by Cai et al.^19^ It should firstly be noted that the positioning of the Si element is not the same. In the aforementioned work, the Ta atom was replaced with a Si dopant, but in the current investigation, the O atom is replaced by the Si dopant. Furthermore, the study conducted by the Cai et al. focused on the λ-Ta_2_O_5_ phase, while our current research investigates the Si-doped features inside the $\epsilon$-Ta_2_O_5_ structure. Several experimental^55,74^ and theoretical investigations^58,75–77^ have shown that the substitution of the O atom in $\epsilon$-Ta_2_O_5_ with a N dopant may lead to significant alterations in its electrical and optical characteristics. Based on the findings of prior experimental and theoretical investigations, we have chosen to examine the impact of doping by replacing O atoms with Si in the $\epsilon$-Ta_2_O_5_ structure. When investigating the RS of Ta_2_O_5_-based memristor, it is important to consider the ${E}_{fe}$ of OVs, the conductivity of the Ta_2_O_5_ layer, and the interaction of dopants with OV, as these factors can affect the stability and regularity of CFs in the device^78,79^. The calculated energy gaps between defect state and conduction band of Si-doped and combined Si-doped with OV are 0.73 eV, 0.584 eV, respectively. As can be seen in Fig. 6, with the creation of OV in Si-doped Ta_2_O_5_, the PDOS shows broadened and increased peaks in the vicinity of the Fermi level, indicating the presence of defect states that can increase the conductivity of the material.

**Figure 7:**
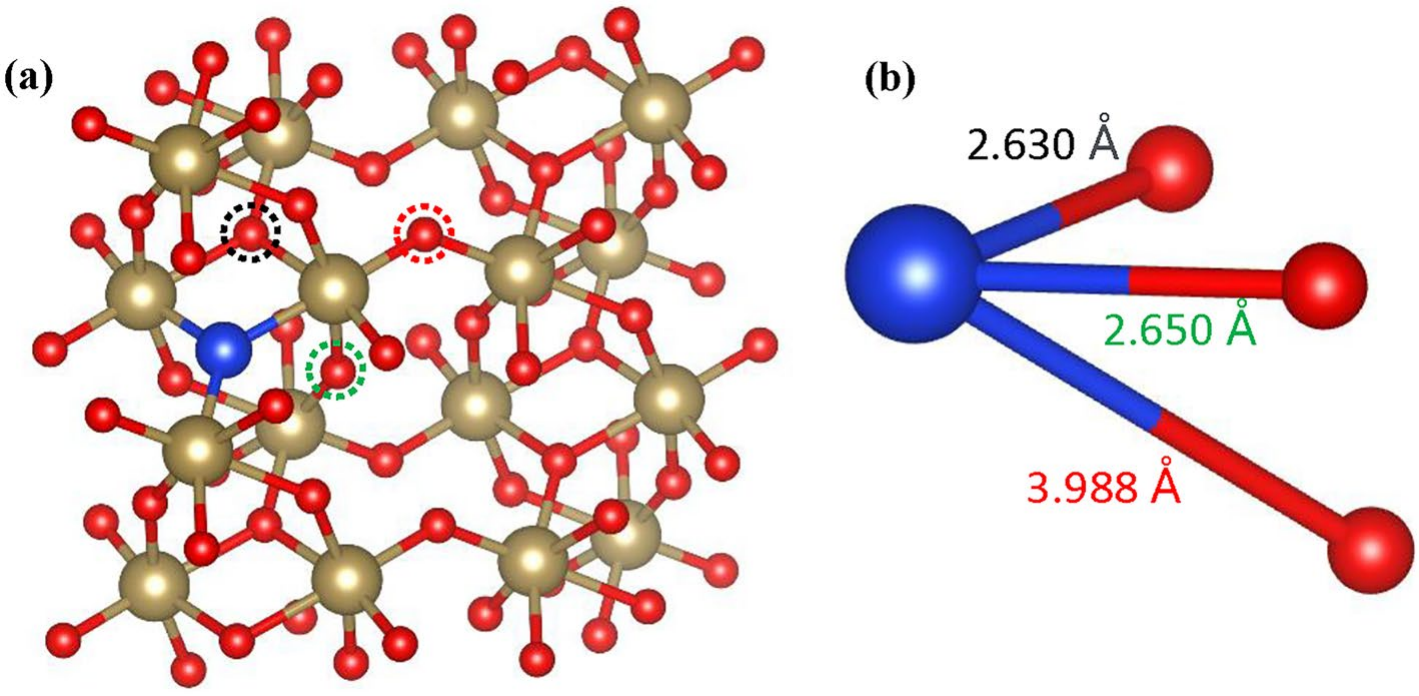
(**a**) Si-doped $\epsilon$-Ta_2_O_5_ structure combined with oxygen vacancies. Different colors in the dotted circles represent different positions of the O atom with respect to the Si atom. The blue, tan, and red spheres denote the Si, Ta, and O atoms, respectively. (**b**) Distances of O atom from Si atom for different sites.

Additionally, the Fermi energy increases and shifts towards the conduction band, which further enhances the conductivity. Furthermore, it is observed that the calculated formation energy for single O$\epsilon$V in Si-doped $\epsilon$-Ta_2_O_5_ reduces to a lower value of 4.12 eV, which may cause the reduction in SET/RESET voltage in Si-doped Ta_2_O_5_-based memristor^80^. The presence of the silicon dopant should also have a stabilizing effect providing consistency on the OVs in the vicinity of those O atoms. To check this, we have analyzed the ${E}_{fe}$ of OVs at different positions of Si doped Ta_2_O_5_. The O atoms at three locations are removed and corresponding ${E}_{fe}$ are calculated, as shown in Fig. 7. The obtained formation energies are 4.12 eV, 5.05 eV, and 5.78 eV for the three different distances of 2.630 Å, 2.650 Å, and 3.988 Å, respectively. We observed that the O atoms located near to the Si impurity have a comparatively low ${E}_{fe}$ of OVs. These findings fit the previous research well^19^. This is likely due to the influence of the Si dopant on the electronic structure and bonding environment of the neighboring oxygen atoms. We have calculated the interaction energy, ${E}_{interaction}$ between the Si dopant and OV using the relation as ${E}_{interaction}= {E}_{SiOV} {-E}_{separate}$. Here, ${E}_{SiOV}$ denotes the energy of the combined Si-doping and OV and ${E}_{separate}$ denotes the energy of the separate defect generated by the Si atom and OV. Our calculation showed that the interaction energy between the Si atom and OV is ~ 2.20 eV, demonstrating a significant attraction between OV and Si atom. Hence, OV develops in the vicinity of the Si atom, which is advantageous to the uniformity of CFs produced by OVs. This phenomenon is in line with the capacity of the Si dopant to impede the stochastic generation of CFs triggered by OVs^19^. The DFT simulations present here provide insight into performance trends, which is valuable in assisting in material selection and optimization for improved switching, guiding experimental efforts.

The determination of the doping characteristics of a defect relies heavily on the computation of the charge state of the point defect. Nevertheless, we believe that the issue of the OV charge state in memristors is more complex than the typical formation energy and transition level calculations for the single-point defect, which are often carried out for dopant analysis. The most stable charge state of OV should vary with the local environment because the formation energy of charged defects is a function of Fermi level, whereas that of neutral defects is not. We may determine the charge state of OV assuming an isolated single defect. Then, the initial charge state of OV calculated shall dictate the transport of single OV under external electrical bias. Nevertheless, the charge state of OV may change further during the course as the local concentration of OV varies along with Fermi level to form a filament where the OV charge state should return to neutral. Due to the greater complexity of the function of OV in memristors compared to dopants in semiconductors, we posit that a distinct examination of the charge state of OV in memristor applications is merited.

## Conclusions

In conclusions, the electrical transport characteristics of $\epsilon$-Ta_2_O_5_ and λ-Ta_2_O_5_ polymorphs have been thoroughly examined using density functional theory calculations. Boltzmann transport theory is used to determine the vacancy-induced electrical conductivity of both structures. Creation of vacancy defects in O$\epsilon$, O_2f_, and O_3f_ coordinated sites generate localized mid-gap defect states within the energy bandgaps. In contrast, the introduction of the O_IL_V defect results in a semiconductor-to-metal transition in Ta_2_O_5_, leading to an improved electrical conductivity. The O_IL_V also shows the lowest formation energy, indicating the lower voltage required for SET/RESET process for this type of vacancy. The introduction of OVs leads to a shift in the Fermi level towards the conduction band for all types of OVs, which can aid in electronic transport and boost conductivity. Further, the formation energy is found to be lowered in Si-doped Ta_2_O_5_, which depends on the OV location, with the lowest energy found for the OV located closest to the Si atom- this appears to be advantageous to the uniformity of CFs produced by OVs. These results can act as guidelines for additional experimental work aimed at improving the regularity and switching characteristics of RS for Ta_2_O_5_-based resistive random-access memory.

### Supplementary Information

Supplementary Figures.

## Data Availability

The datasets used and/or analyzed during the current study available from the corresponding author on reasonable request.
