# Supplementary material for: Effect of oxygen vacancy and Si doping on the electrical properties of Ta2O5 in memristor characteristics
Source: Sci Rep. 2023 Oct 3;13:16656. doi: 10.1038/s41598-023-43888-z (PMC10547760; doi:10.1038/s41598-023-43888-z)
Supplement: Supplementary file 1 — Supplementary Figures. [file 41598_2023_43888_MOESM1_ESM.docx]

**Supplementary Information**

**Effect of Oxygen Vacancy and Si Doping on the Electrical Properties of Ta_2_O_5_ in Memristor Characteristics**

Md. Sherajul Islam^1, 2, *^, Jonghoon Lee^1, 3^, Sabyasachi Ganguli^1^, Ajit K Roy^1*^

^1^Materials and Manufacturing Directorate, Air Force Research Laboratory, Wright-Patterson Air Force Base, Dayton, OH, United States

^2^Spectral Energies, LLC, Dayton, OH, United States

^3^ARCTOS Technology Solutions, Dayton, OH, United States

*Corresponding Authors’ Email: [sheraj_kuet@eee.kuet.ac.bd](mailto:sheraj_kuet@eee.kuet.ac.bd); [ajit.roy@us.af.mil](mailto:ajit.roy@us.af.mil)


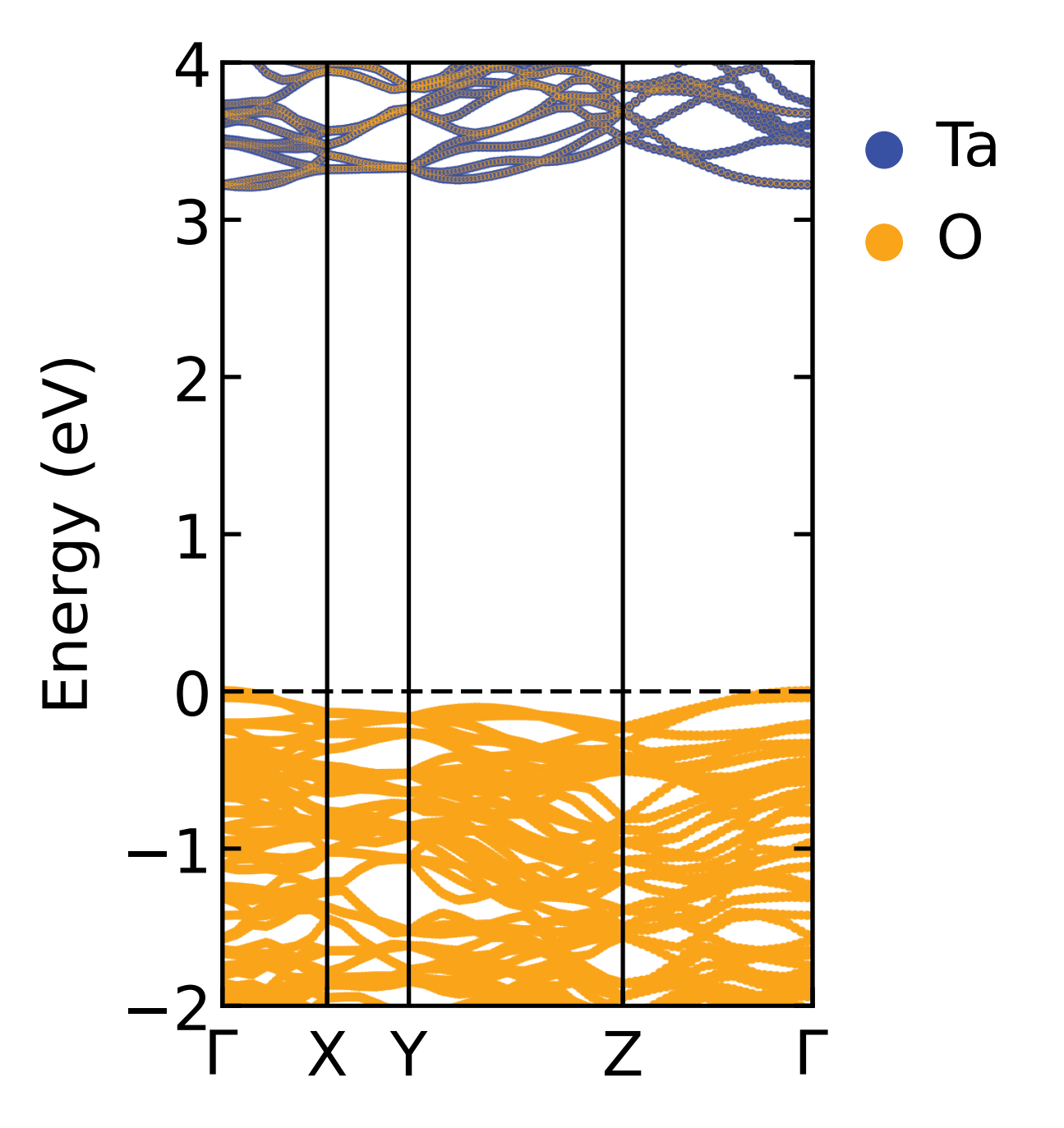

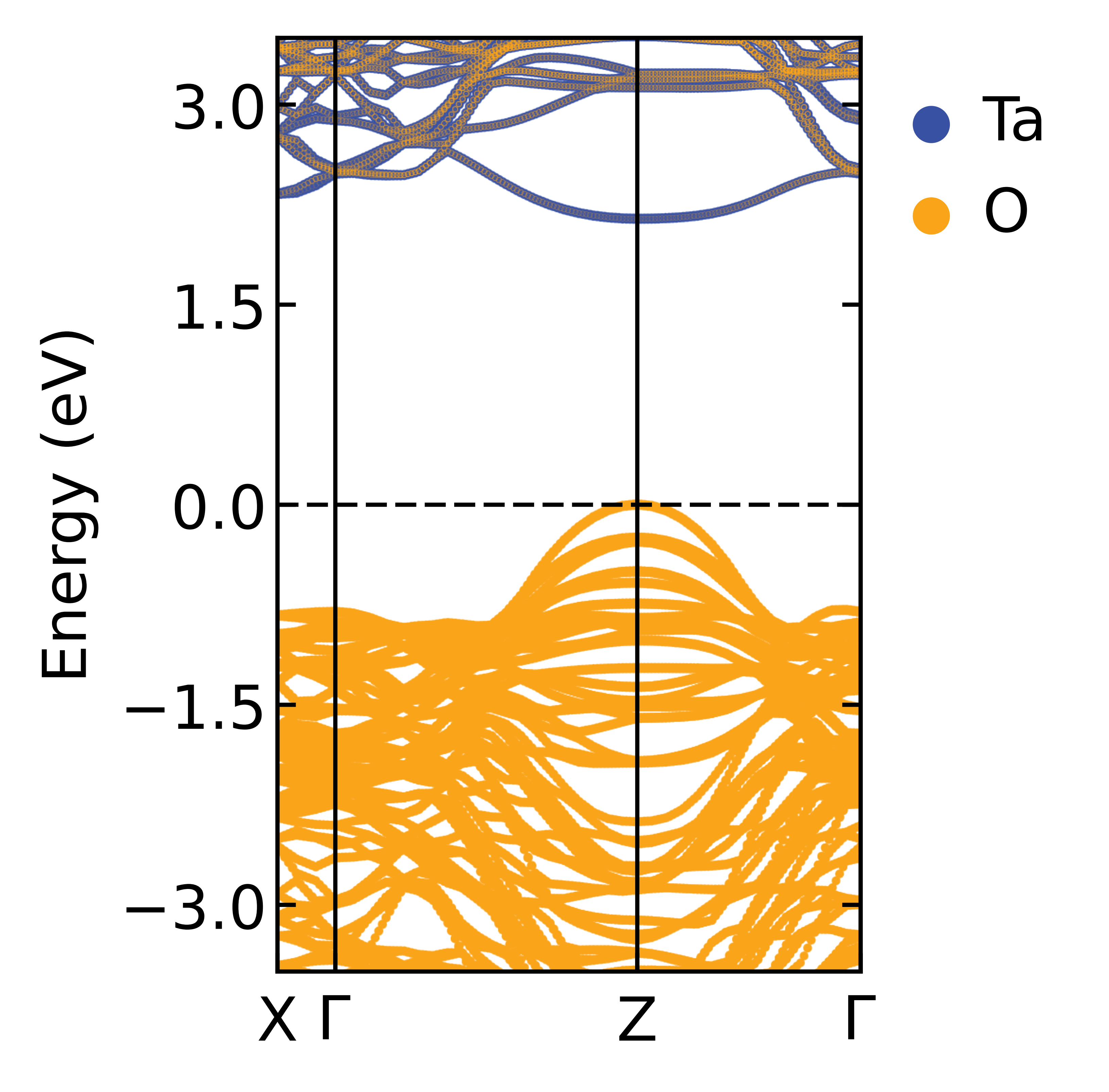


**(b)**

**(a)**

**Figure S1.** Projected electronic band structures for (a) ϵ- and (a) λ-phase pristine Ta_2_O_5_ structures obtained from the PBE_sol_ GGA functional. The reference energy level (dotted line) is touched with the valence band maximum for both structures.


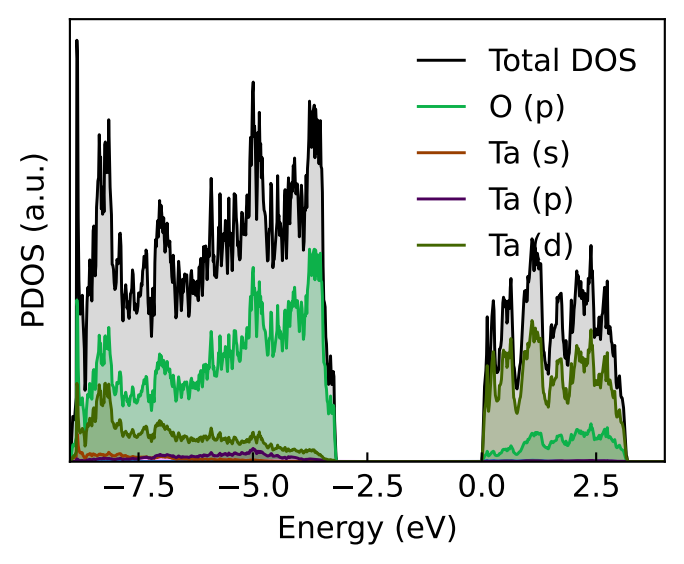

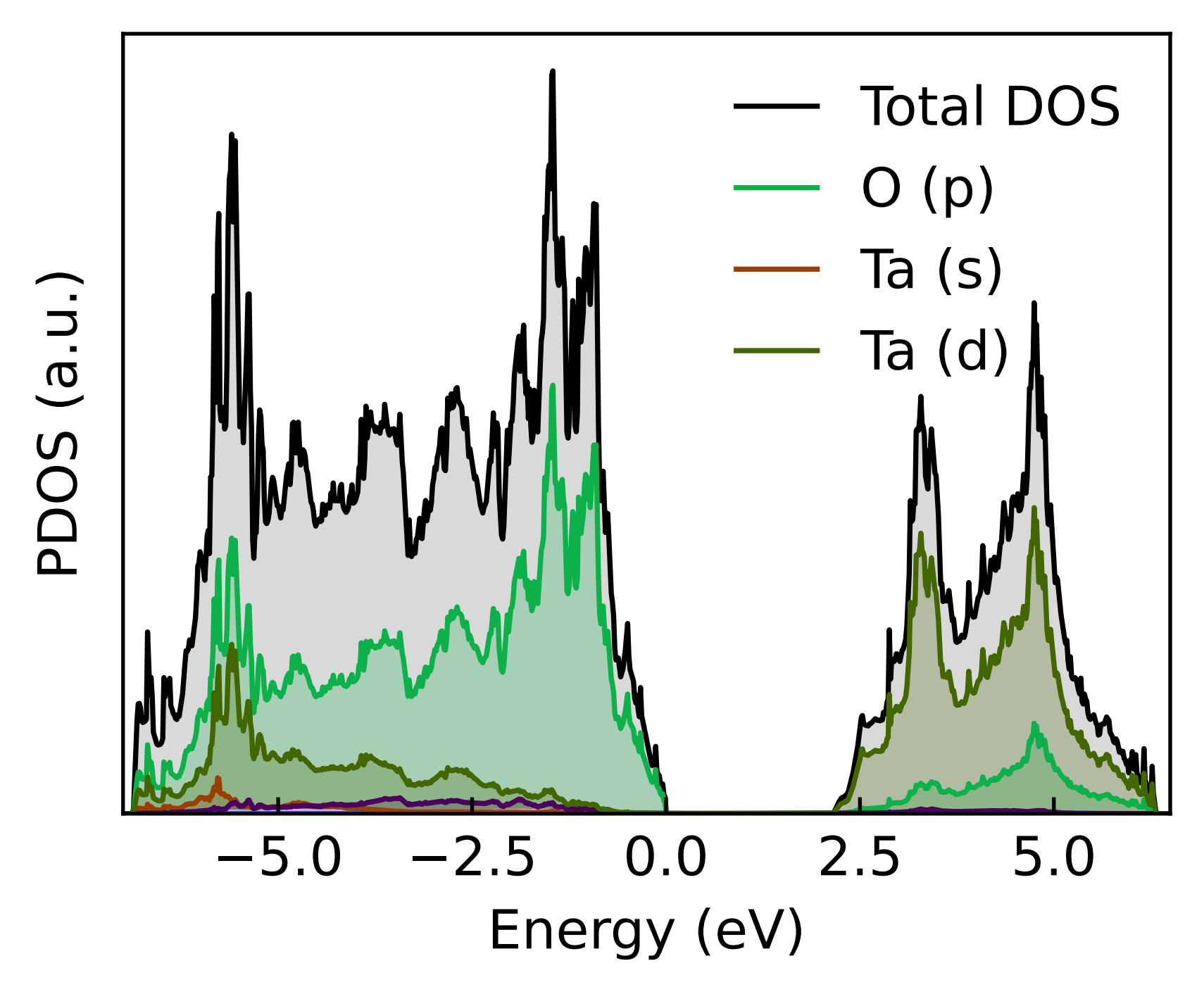


**(a)**

**(b)**

**Figure S2.** Projected density of states (PDOS) for (a) ϵ- and (a) λ-phase pristine Ta_2_O_5_ structures.
